# Arterial oxygenation and mortality in patients admitted to intensive care following out-of-hospital cardiac arrest

**DOI:** 10.1186/2197-425X-3-S1-A204

**Published:** 2015-10-01

**Authors:** R Ahmed, L Paton, P Stenhouse, A Davidson, A Mackay

**Affiliations:** Victoria Infirmary/ NHS Greater Glasgow & Clyde, Critical Care, Glasgow, United Kingdom

## Intr

The effects of hyperoxia at a cellular level are well known, with formation of free radical species causing reactive damage. It has previously been shown that hyperoxia is associated with increased mortality in patients resuscitated from cardiac arrest [1]. However, hypoxia is likewise deleterious to outcomes in this patient cohort.

## Objectives

We sought to examine whether mortality varies with arterial oxygenation in the first 24 hours after admission to our intensive care unit (ICU) following out of hospital cardiac arrest (OOHCA).

## Methods

This was a retrospective, consecutive case series of patients admitted to the ICU at the Victoria Infirmary, Glasgow following OOHCA over a 15 year period between 1999 and 2014. Patients were identified and characterised using the WardWatcher™ database. Three subgroups were generated based on the arterial partial pressure of oxygen (P_a_02) provided as part of severity scoring: hyperoxia was defined as P_a_02 > 15, normoxia as P_a_02 10-15 and hypoxia as P_a_02 < 10 kPa. Hospital mortality was the primary outcome measure and statistical significance was assessed using Fisher's exact test.

## Results

There were 124 patients (73 men, 58.9%) with a median age of 64 years (IQR 52-70 years) admitted following OOHCA. For the 97 patients with complete severity scoring, the median APACHE II score was 31 (IQR 24-35) and mortality prediction 80.3% (IQR 59.4%-87.9%). Survival to hospital discharge was achieved in 29 patients (23.4%), yielding a standardised mortality ratio of 0.95. Median hospital length of stay was 16 days (IQR 8-27 days). Data on arterial oxygenation were available for 103 patients.

**Table 1:** Prevalence & mortality in art. oxygenation subsets.

	Number (%) of patients	Hospital mortality
Hyperoxia	71 (68.9%)	74.6%
Normoxia	20 (19.4%)	75.0%
Hypoxia	12 (11.7%)	83.3%

There was no statistically significant difference in hospital mortality between groups (P = 1.0 for hyperoxia vs. normoxia; P = 0.68 for normoxia vs. hypoxia).

## Conclusions

We demonstrated survival to hospital discharge in almost a quarter of patients admitted to ICU post-OOHCA. Despite international resuscitation guidelines to the contrary [2], a significant proportion of our patients continue to be exposed to hyperoxia. However, there is no evidence that arterial oxygenation levels impact outcomes in our unit.
